# Gritty sensation on catheter: A new clinical sign for self-diagnosis of stone formation in the continent urinary pouch

**DOI:** 10.4103/0970-1591.60457

**Published:** 2010

**Authors:** Apul Goel, Anuj Goel, Diwakar Dalela, S. N. Sankhwar

**Affiliations:** Department of Urology, CSM Medical University, Formerly King George Medical University, Lucknow - 226 003, India,; 1 Wellcome Trust Center for Human Genetics, University of Oxford, Oxford, UK

**Keywords:** Bladder substitution, continent cutaneous pouch, self-catheterization, stone

## Abstract

With more experience and better management, the incidence of complications like stone formation after continent urinary diversion is uncommon today. We report a case of large stone bulk in a patient who underwent this surgery 10 years back and who suggested the formation of stones in the pouch herself by sounding them. Proper counseling, regular pouch irrigation and follow-up are essential in any kind of diversion.

## INTRODUCTION

Occasionally, urinary diversion is needed in cases of complex vesicovaginal fistula who have failed repeated repairs. We report one such case that underwent continent urinary diversion and reported 10 years later when she noticed a gritty sensation on passing catheter that was later found to be due to a large stone bulk in the pouch.

## CASE REPORT

**Figure 1:**
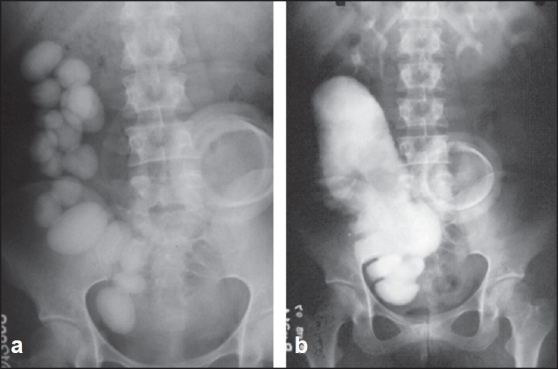
Plain X-ray abdomen (a) shows multiple stones in the right side of the abdomen. Intravenous urogram (b) shows well-preserved kidneys with large reservoir and stones. Circular shadow of colostomy device is also seen

A 20-year-old woman presented with vesicovaginal (VVF) and rectovaginal fistula (RVF) due to obstructed labor. She underwent a temporary transverse colostomy followed by two failed attempts of VVF repair. One year later, the RVF was repaired successfully by the vaginal route and a simultaneous continent cutaneous urinary diversion procedure (right colon pouch with Mitrofanoff continence mechanism) was performed. She was then lost to follow-up and could not come even for colostomy closure due to poor socioeconomic condition. She presented after 10 years when she noticed a gritty sensation while passing catheter for pouch emptying. On evaluation, she had normal renal functions, with serum creatinine of 1.0 mg%, while an intravenous urogram revealed a large pouch full of stones [Figure 1]. In view of the large stone bulk, she was subjected to open surgical removal of stones with simultaneous colostomy closure. Stone analysis revealed that the stones were composed of calcium, magnesium and ammonium phosphate.

## DISCUSSION

Stone formation is a known complication of all reservoirs.[1–3] This patient belonged to the underprivileged community and could not come for regular follow-up. Moreover, the patient was irregular in performing clean intermittent catherization for pouch empting and was not washing the pouch due to economic reasons. Risk factors for stone formation in such a setting have been well elucidated in the literature, which include persistent infection with alkalinization of urine, alterations of urinary excretion products by the intestine and foreign bodies like sutures.[3] Alterations in the bowel mucosa may also serve as a nidus for stone formation. Additionally, alterations in the intestinal mucus, particularly in the presence of infection or obstruction, may serve as a nidus or, more importantly, may interfere with emptying and thereby exacerbate infection and stone formation.[2] Fortunately, she did not develop any symptomatic infection and hence calculi formation went unnoticed till she self-diagnosed them by sounding.

This case demonstrates that if continent urinary diversion is planned, the patient should be counseled well about the need of regular self-catheterization, pouch irrigation and regular follow-up even if asymptomatic and may be advised to report any abnormal gritty sensation while draining the pouch by catheter. This grittiness may be experienced while passing the catheter or may even be experienced while withdrawing the catheter-sometimes better in the latter situation as the pouch becomes free of liquid urine and stones come close to each other and the catheter within the collapsing pouch. Although the stones were smooth surfaced, the passage or withdrawal of catheter through a significant stone burden can still produce gritty sensation to an intelligent patient.
